# Mamma Mia: Finger Injury in a Basketball Player in the Italian Professional League

**DOI:** 10.7759/cureus.5334

**Published:** 2019-08-07

**Authors:** Eric Yuschak, Stacy Chase, Furqan Haq

**Affiliations:** 1 Family Medicine, St. Petersburg General Hospital, St. Petersburg, USA; 2 Miscellaneous, Hospital Corporation of America West Florida, Tampa, USA

**Keywords:** sagittal band injury, finger tendon injuries, sport medicine

## Abstract

In many professional sports, the dexterity and importance of the player's hands are crucial for optimal importance. The health and treatment of injuries focuses on protection while providing the ability to function. This is a case of a professional basketball player whose team was fighting for a playoff position in the Italian basketball league. Other common finger tendon injuries are also reviewed to help keep a broad differential diagnosis in mind when evaluating patients.

## Introduction

Hand injuries are common in athletics and account for up to 9% of all sports injuries [1]. The sagittal band is the primary stabilizer of the extensor tendon at the metacarpophalangeal (MCP) joint and injuries to this area contribute to the hand injuries seen. It helps prevent bowstringing during MCP hyperextension and resists ulnar deviation of the tendon during MCP flexion. A sagittal band rupture is also known as boxers knuckle can result from both acute and chronic events. Acutely, direct blunt trauma to the MCP joint seen during a boxers punch or seen with forceful resisted flexion or extension can result in the injury. Chronically it occurs from inflammatory conditions such as rheumatoid arthritis where the ulnar deviation of the digits weakens and tears the radial sagittal band. Acute injuries can initially be splinted in an extension splint whereas chronic injuries typically respond better to surgical reconstruction. The middle finger is most commonly affected and seen in approximately 48% of cases because the middle finger MCP is more prominent [2]. The radial side sagittal band is also more commonly affected than ulnar sided seen in a 9:1 ratio [3].

The injury is classified using the Rayan and Murray classification system. It is divided into three groups. The first group is a sagittal band injury without extensor tendon instability. The second stage involves extensor tendon subluxation. And finally, the third stage involves extensor tendon dislocation. Ultrasound can be a useful diagnostic tool when swelling obstructs physical exam. In an unpublished lecture at the 2019 American Medical Society for Sports Medicine Annual Meeting Dr. Greditzer, a musculoskeletal radiologist, said by using ultrasound the extensor tendon subluxation on MCP flexion can be visualized in real time demonstrating the value of having an ultrasound readily available in office in addition to more accurate injections.

## Case presentation

This case is of a 27-year-old African American, right hand dominant male professional basketball player who experienced right middle finger pain and swelling after an injury during competition several days prior. He said he was attempting to grab the ball away from an opponent when he got tangled up and experienced blunt trauma to his hand. He immediately felt a sharp pain. He denied obvious dislocation or deformity of his hand. Since that time he said the swelling and pain have persisted and he rated it a 6/10 in severity. He admitted to a snapping sensation at his knuckle with flexion of his finger. He tried ice and anti-inflammatory medication with some relief. He denied numbness and tingling of his hand. He denied loss of strength but had limited active range of motion with flexion of his middle finger of his right hand. His team’s athletic trainer had placed him in a splint to limit flexion so that he could continue to play basketball as his team was attempting to make the playoffs. He denied significant past medical history. He has had a sports hernia surgery several years ago but denied other surgeries. He has injured his other hand before and said this is similar. At that time, he was told he had a sagittal band rupture which was managed non operatively. His mother, father and one brother are living and healthy. He denies tobacco or recreational drugs and drinks socially.

The initial differential diagnosis for an acute athletic finger injury includes fracture and flexor or extensor injuries such as sagittal band rupture, jersey finger, mallet finger, and boutonniere deformity.

**Figure 1:**
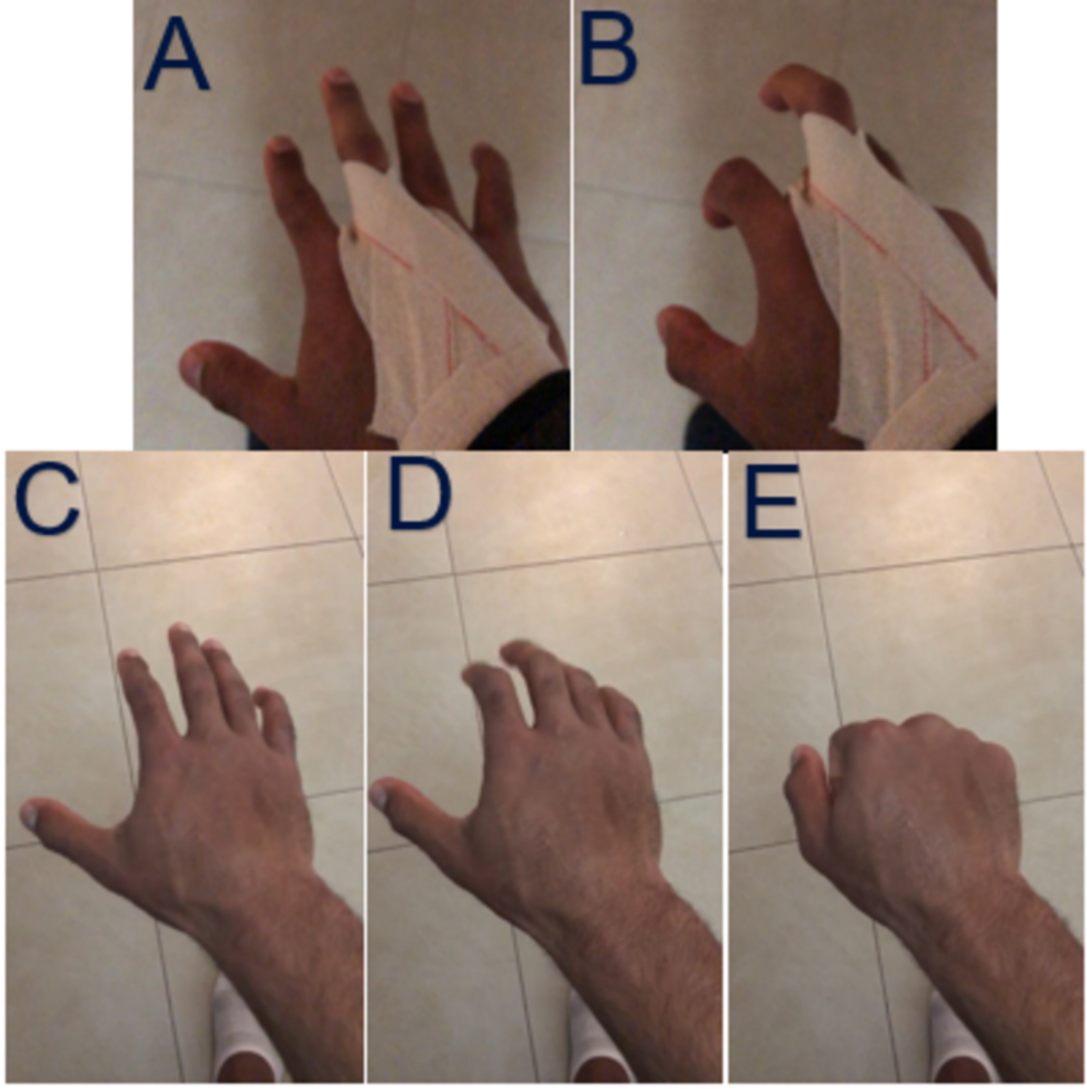
Hand Photographs A, B) demonstrates the extension splint used to limit metacarpophalangeal (MCP) flexion; C, D, E) demonstrate increased motion without reported snapping sensation. Swelling is still pronounced and middle finger MCP prominence is demonstrated in panel E which exposes this finger to this injury most often.

The workup and treatment of the player included immediate evaluation from the athletic training staff. Radiographs were obtained and negative for osseous abnormalities. He continued conservative management and finger extension splinting to limit the amount of flexion. The goal of the splint was to allow maximum 30 degrees of flexion at the metacarpophalangeal (MCP) joint. He underwent physical therapy and at two weeks his motion was improved and did not experience the snapping sensation anymore. He was able to continue to compete with his splint. Figures 1A-1B demonstrate the extension splint used to limit MCP flexion. Figures 1C-1E were taken during follow up and demonstrate increased motion without reported snapping sensation. Swelling is still pronounced and middle finger MCP prominence is demonstrated in panel E which exposes this finger to this injury most often.

The final diagnosis for the player based on the location of the injury, negative radiographs, and symptoms of swelling and initial snapping sensation was a sagittal band rupture.

## Discussion

Another traumatic extensor injury is a mallet finger. It is caused by disruption of the terminal extensor tendon distal to the distal interphalangeal (DIP) joint seen during traumatic impaction (sudden forced flexion) or possibly a dorsal sided laceration. Symptoms include pain and swelling at the DIP joint. The fingertip rests at 45 degrees of flexion and lacks active DIP joint extension. The injury is classified using the Doyles system which described four groups of injuries [4]. The first is a closed injury with or without a small dorsal avulsion fracture. The second is an open injury from a laceration. The third is an open injury that involves loss of skin and tendon substance. And finally, the forth is a mallet fracture and is subdivided into A involving physeal fracture in pediatrics, B involving 20%-50% of the articular surface and C involving greater than 50% the articular surface. The management can be both extension splinting of the DIP joint or surgical.

Boutonniere deformity is an extensor tendon injury that is differentiated from mallet finger based on location. A boutonniere deformity is a zone three injury, more proximal, caused by rupture of central slip over the proximal interphalangeal (PIP) joint leading to PIP joint flexion and DIP joint extension [5].

Jersey finger is another common acute finger injury but is a flexor injury. This occurs from an avulsion of the flexor digitorium profundus (FDP) from the insertion at the base of the distal phalanx. It is considered a zone one flexor tendon injury which is the most distal zone. In this injury, the ring finger is most commonly involved cases because the ring finger experiencing the greatest average force during a grasp and pull away which is the common complaint of athletes experiencing this [4,6]. Symptoms can include pain and tenderness over the volar distal finger. The patient holds the finger in slight extension and has no active flexion at the DIP joint which is the opposite position seen from mallet finger where the finger is once again held in flexion and lacks DIP joint extension [7]. This is a surgical condition with prolonged healing time. It is classified using the Leddy and Packer system which described five groups of injuries. The first is an FDP tendon retraction to the palm. The second is an FDP retraction to the level of the PIP joint. The third is a large avulsion fracture that limits retraction to the level of the DIP joint. The forth has an osseous fragment and simultaneous avulsion of the tendon from the fracture fragment and is considered a “double avulsion.” And finally, the fifth group is a ruptured tendon with bone avulsion with bony comminution of the remaining distal phalanx.

Finally, the trigger finger is a stenosis tenosynovitis from flexor tendon sheath inflammation. Although it is typically more chronic in nature, patients present with finger clicking and pain at distal palm near the A1 pulley that overlies the metacarpal phalangeal joints [8]. The finger may become locked in a fixed position which could make it confused with a more acute traumatic finger injury. It is most common in the ring finger of diabetics. It is classified using the Green system which breaks it into four groups [8]. The first is palm pain and tenderness at the A1 pulley. The second involves catching of the involved digit. The third is locking of the digit passively but correctable. And finally, the fourth group involves a fixed locked digit. Treatment can be non-operative with night splinting, activity modification and anti-inflammatory medications. Steroid injections can also be used. Surgical intervention may also be indicated and involved A1 pulley release.

## Conclusions

Professional athletes are a special population of patients. The treatment of injuries in this population is important. This case demonstrates the adaptions the player and training staff could make so that the player could safely continue to compete without jeopardizing their health and skill set for future employment or playing opportunities. In addition to recognizing the needs of the athlete population, the differential diagnosis of common finger tendon injuries was reviewed. The hand is a complex region with many different bones, ligaments, and tendons; a basic understanding of commonly seen injuries is crucial for patient care at the primary care level.
